# Retraction: Enhancing IoT cybersecurity through lean-based hybrid feature selection and ensemble learning: A visual analytics approach to intrusion detection

**DOI:** 10.1371/journal.pone.0346538

**Published:** 2026-04-06

**Authors:** The PLOS One Editors

The *PLOS One* Editors retract this article [1] due to concerns about compromised peer review and compliance with PLOS policy on Authorship. We regret that the issues were not addressed prior to the article’s publication.

In addition, the article cited as reference 16 in [1] was retracted before this article was published.

IZ, EO, SJ, HA, and AA did not agree with the retraction. SA, SH, and NP either did not respond directly or could not be reached.
